# The Detection of Blood in Excreta

**Published:** 1907-04-20

**Authors:** 


### Points in Diagnosis.

### THE DETECTION OF BLOOD IN EXCRETA.

### Small Quantities of Blood in Vomited Matter, and in Fæces.

When the amount of blood in the stomach contents or in the stools is large, there is little difficulty in its detection. It is usually obvious to the naked eye. In connection with the stools there may be difficulty in cases of true melæna from gastric or duodenal hæmorrhage; for the patient may be taking charcoal, iron, or bismuth, each of which may render the motions black, and one may be in doubt as to whether the melæna is due to drugs or to altered blood. The doubt in such cases may readily be set at rest by collecting a small quantity of the fæces on a piece of lint, dropping three drops of alcoholic solution of guaiacum resin on to one edge of the black mass, and then pouring on some ozonic ether; when the melæna is due to blood the typical blue colour appears, just as it does in the similar test for blood in urine. This blue colour is due to oxidation of the guaiacum resin, such oxidation not being effected by blood alone, nor by ozonic ether alone, but readily produced by blood and ozonic ether together. The test is a poor one from the chemical point of view, for many other oxidising substances besides blood are able to augment the action of ozonic ether in this way, and so to produce the blue colouration with guaiacum; but from the clinical point of view the test is fairly conclusive, because blood is the only one of these substances likely to be present in the stools. If one wanted to be more accurate still, one would make a solution from the black fæces, and use the spectroscopic test for the detection of the pathognomonic absorption bands of hæmoglobin or hæmatin.

#### Where Examination is Desirable.

It is when the amount of blood is too small to be obvious to the naked eye that its detection requires special care. The presence of small quantities of blood in the gastric contents may be of great importance, for example, in distinguishing cases of carcinoma of the stomach from cases of edentalous dyspepsia. Every medical man is constantly coming across patients in whom he wonders whether the gastric pains and vomiting are really due to neoplasm or whether they may not be entirely due to the inefficient action of teeth which are either carious and useless, surrounded by sepsis, or are deficient in number, the few remaining fangs being worse than useless, in that they are themselves too few to properly masticate the food, whilst at the same time they prevent the jaws from closing upon the food. Profuse hæmorrhage is distinctly rare in cases of gastric carcinoma; the "coffee ground" vomit, in which the coffee-ground appearance is due to small quantities of altered blood, is by no means common. The majority of cases of cancer of the stomach present no blood in the vomit, recognisable by the naked eye. In such cases the method of testing for blood to be described below may materially assist the diagnosis.

In the case of stools, again, small quantities of blood may escape detection in the ordinary way, and yet may be discovered by a simple procedure; the kind of case in which this may be very useful is one of tuberculous ulceration of the intestine. In the majority of such cases there is neither diarrhœa nor obvious blood with the stools; quite often the autopsy in a case of phthisis shows extensive tuberculous ulceration of the bowel when there was little or no clinical evidence of it during life. The detection of repeated small quantities of blood in the stools, apart from hæmoptysis, would materially assist the diagnosis. The same applies to cases of diarrhœa, in which acute or ulcerative colitis may be suspected; and other similar conditions readily occur to one.

#### The Method.

The method is as follows:—About two drachms of the filtered stomach-contents, or a similar quantity of the fæces made up into a thin paste with ordinary tap water,* are poured into a large test-tube, together with about a third-part of ordinary B.P. acetic acid. The mixture is well shaken, and then ether is added, the amount of ether required being equal to about half the total bulk of the fluid previously in the tube. The whole is then well shaken up together. The ether takes up most of the blood. The test-tube is then allowed to stand for a minute or two until its contents have separated into two layers; the clear ether containing the blood floats uppermost and should be poured off into a fresh clean test-tube; on now adding to it a drop or two of alchoholic solution of guaiacum resin and about half a drachm of ozonic ether, or 20 drops of a 5-per-cent. solution of peroxide of hydrogen, the typical blue colour will appear if blood is present. A similar ethereal extract may be examined spectroscopically if preferred.

When the blood is extracted in this way by ether after acidification of the stomach-contents or fæces with acetic acid, it is said that as little as one milligramme of blood can be detected. The test, of course, does not indicate the source of the blood, and to prevent any fallacy arising from the small amount of blood there is in butcher's meat, it is as well to have given the patient a diet containing no blood for a day or two beforehand. Blood swallowed from a source in the mouth, nose, or lungs will have to be excluded upon clinical grounds before the positive reaction can be taken as evidence of a bleeding focus in the stomach; and in regard to the stools the blood may occasionally be extraneous, as, for example, during menstruation. Nevertheless, the test is itself so easy that there are many cases in which the practitioner will gladly apply it. The diagnosis may often be assisted by the detection of blood which is not evident to the naked eye.

* The tap water should always have been previously tested with the guaiacum and ozonic ether, for it not infrequently happens that the tap water by itself contains substances which give the blue colouration.

